# Beating the odds: Sustained Chagas disease vector control in remote indigenous communities of the Argentine Chaco over a seven-year period

**DOI:** 10.1371/journal.pntd.0006804

**Published:** 2018-10-02

**Authors:** M. Sol Gaspe, Yael M. Provecho, María P. Fernández, Claudia V. Vassena, Pablo L. Santo Orihuela, Ricardo E. Gürtler

**Affiliations:** 1 Universidad de Buenos Aires. Facultad de Ciencias Exactas y Naturales, Laboratorio de Eco-Epidemiología, Ciudad Universitaria, Buenos Aires, Argentina; 2 Consejo Nacional de Investigaciones Científicas y Técnicas-Universidad de Buenos Aires. Instituto de Ecología, Genética y Evolución de Buenos Aires (IEGEBA), Ciudad Universitaria, Buenos Aires, Argentina; 3 Coordinación de Vectores, Dirección Nacional de Epidemiología y Análisis de la Situación de Salud, Ministerio de Salud de la Nación, Buenos Aires, Argentina; 4 Earth Institute, Columbia University, New York, New York, United States of America; 5 Centro de Investigaciones de Plagas e Insecticidas (UNIDEF, CITEDEF, CONICET, CIPEIN), Buenos Aires, Argentina; 6 Cátedra de Química Analítica Instrumental, Facultad de Farmacia y Bioquímica, Universidad de Buenos Aires, Buenos Aires, Argentina; 7 Departamento de Investigación e Ingeniería Ambiental (3iA), Universidad Nacional de San Martín, Buenos Aires, Argentina; Universidade Federal do Rio de Janeiro, BRAZIL

## Abstract

**Background:**

Rapid reinfestation of insecticide-treated dwellings hamper the sustained elimination of *Triatoma infestans*, the main vector of Chagas disease in the Gran Chaco region. We conducted a seven-year longitudinal study including community-wide spraying with pyrethroid insecticides combined with periodic vector surveillance to investigate the house reinfestation process in connection with baseline pyrethroid resistance, housing quality and household mobility in a rural section of Pampa del Indio mainly inhabited by deprived indigenous people (Qom).

**Methodology/Principal findings:**

Despite evidence of moderate pyrethroid resistance in local *T*. *infestans* populations, house infestation dropped from 31.9% at baseline to 0.7% at 10 months post-spraying (MPS), with no triatomine found at 59 and 78 MPS. Household-based surveillance corroborated the rare occurrence of *T*. *infestans* and the house invasion of other four triatomine species. The annual rates of loss of initially occupied houses and of household mobility were high (4.6–8.0%). Housing improvements did not translate into a significant reduction of mud-walled houses and refuges for triatomines because most households kept the former dwelling or built new ones with mud walls.

**Conclusions/Significance:**

Our results refute the assumption that vector control actions performed in marginalized communities of the Gran Chaco are doomed to fail. The larger-than-expected impacts of the intervention program were likely associated with the combined effects of high-coverage, professional insecticide spraying followed by systematic vector surveillance-and-response, broad geographic coverage creating a buffer zone, frequent housing replacement and residential mobility. The dynamical interactions among housing quality, mobility and insecticide-based control largely affect the chances of vector elimination.

## Author summary

Efforts to prevent the transmission of the parasite that causes Chagas disease have been directed at eliminating its insect vector species from human dwellings via insecticide applications. The outcome of these interventions has usually been measured through vector-related metrics in prospective studies ranging up to a few years. Longer-term intervention studies that additionally monitor other social determinants of house infestation, such as housing quality and population movement, are lacking. Our seven-year study addresses this gap in a remote rural area of the Argentine Chaco inhabited by deprived indigenous communities. We show sustained vector control headed towards local elimination despite the highly adverse social context and the occurrence of moderate pyrethroid resistance in vector populations. Housing-quality dynamics and household mobility displayed complex patterns that may affect domestic triatomine populations. Household mobility within the area was intense: movers usually torn down their previous precarious houses and built new, equally precarious houses. Housing dynamics included structural improvements, disappearance and construction of new dwellings elsewhere within the study area. We infer that these eco-bio-social factors, including the broad geographic coverage of sustained vector control creating a peripheral buffer zone, substantially increased the long-term effectiveness of the intervention program.

## Introduction

Neglected tropical diseases (NTDs) stem from the complex interactions among social, economic, political, cultural and environmental determinants acting at various temporal and spatial scales [1]. These complex interactions explain why some groups of people, including indigenous communities and the rural poor, are most affected by NTDs and frequently suffer co-infections as part of the cycle of poverty [2,3]. A case in point is the Gran Chaco ecoregion, including sections of Argentina, Bolivia and Paraguay home to numerous indigenous peoples [4–6]. This NTD hotspot is characterized by high prevalence rates of human infection with *Trypanosoma cruzi* (Chagas disease), geo-helminthiases, and unsatisfied basic needs [7].

High levels of house infestation with *Triatoma infestans*, historically the main vector in the Southern Cone countries, are still present in sections of the Gran Chaco despite of the multiple insecticide-based control campaigns conducted over nearly 70 years [5,8–13]. In the absence of effective vaccines and given the limitations of current drugs for massive chemotherapy, Chagas disease prevention efforts have historically relied on residual insecticide spraying and screening of blood donors. Vector control campaigns contracted the geographic range of *T*. *infestans* [14–16] and suppressed the domestic transmission of *T*. *cruzi* mediated by *T*. *infestans* in various countries and provinces since the mid-1990s [6,15]. However, persistent house reinfestation after insecticide application fueled by peridomestic foci in different areas of the Argentine Chaco [17], combined with growing evidence of the existence of sylvatic foci of *T*. *infestans* [18–21] and the emergence of pyrethroid insecticide resistance (reviewed in [22]), cast doubts on how feasible is to achieve the elimination of *T*. *infestans* in the Gran Chaco [23], one of the initial goals of the Southern Cone Initiative in 1991. Lack of consistent state policies and of a sustainable vector surveillance-and-response system in resource-constrained rural areas, among other factors, contribute to a persistent or recurrent public health issue [4].

The widespread problem of native, highly competent triatomine species that reinfest insecticide-treated dwellings (e.g., [24]) should be addressed by an integrated vector management strategy [25] that considers the eco-bio-social determinants of health vulnerability [26] and house infestation with triatomines [27–29]. Two major components of such strategy are community participation and housing improvement. Community participation may assist in the design and implementation of locally adapted, sustainable and more effective vector control strategies, especially in remote, deprived areas [30]. Housing improvement [25] tends to reduce triatomine infestation in domestic and peridomestic structures [29,31–36]. The historical trend toward rural housing improvement, albeit at a widely different pace across regions and countries, suggest any assessment of the effectiveness of vector control actions should also encompass structural determinants of infestation such as housing quality, type of occupancy, and host availability over time.

As part of a longitudinal program on the eco-epidemiology and control of *T*. *infestans* and Chagas disease in the Argentine Chaco, we detected higher-than-expected post-spraying house infestation rates and apparent residual foci related to moderate (incipient) pyrethroid resistance in two large rural sections (denominated Areas I and II) of Pampa del Indio having 400–500 houses each [37,38]. A residual focus is defined as a post-spraying infestation derived from triatomines that survived the insecticide spraying at house level. These patterns suggested that similar rates of house reinfestation would occur in another large rural section (Area III) where the same community-wide insecticide spraying interventions were simultaneously implemented. All these rural areas had poor housing conditions suitable for triatomines, and high infestation with *T*. *infestans* before control actions. Seroprevalence of human *T*. *cruzi* infection averaged 30% [39]. Two of the rural sections were mainly inhabited by an indigenous people (Qom) that displayed intense residential mobility, especially within Area III [40]. Unexpectedly, the intervention there exerted immediate impacts on house infestation as revealed by timed-manual searches; the few foci detected after blanket insecticide spraying were mainly assigned to external sources as determined by wing geometric morphometry [41]. Identifying processes and mechanisms underlying the successful vector control status achieved in Area III is relevant to the goals of suppressing the major vectors of Chagas disease and interrupt domestic transmission, in concert with the Sustainable Development Goals and the London’s Declaration on NTDs [42].

Here we extend the follow-up of Area III to further investigate the house reinfestation process in connection with baseline pyrethroid resistance, housing dynamics (construction, destruction, abandonment and improvement) and household mobility over a seven–year period. Instead of assuming static environmental conditions and focusing only on vector-related issues, we adopted a more systemic approach and analyzed other household-level, time-variable processes related to the dynamics of housing quality (habitat) and residential mobility, which also affects host availability. Population movement is relevant to the control of NTDs and other infectious diseases in a context-specific manner [43], but the very few studies that examined this subject in connection with Chagas disease vectors did it at a village level [44] or in urban-type settings [45–46]. Our study provides evidence of sustained vector control despite moderate pyrethroid resistance in *T*. *infestans* populations; corroborates the virtual absence of post-spraying foci and the house invasion of other four triatomine species through community-based surveillance, and links the lack of post-spraying foci to frequent housing replacement, residential mobility, and broad geographic coverage of sustained vector control.

## Materials and methods

### Study area

**Fig 1:**
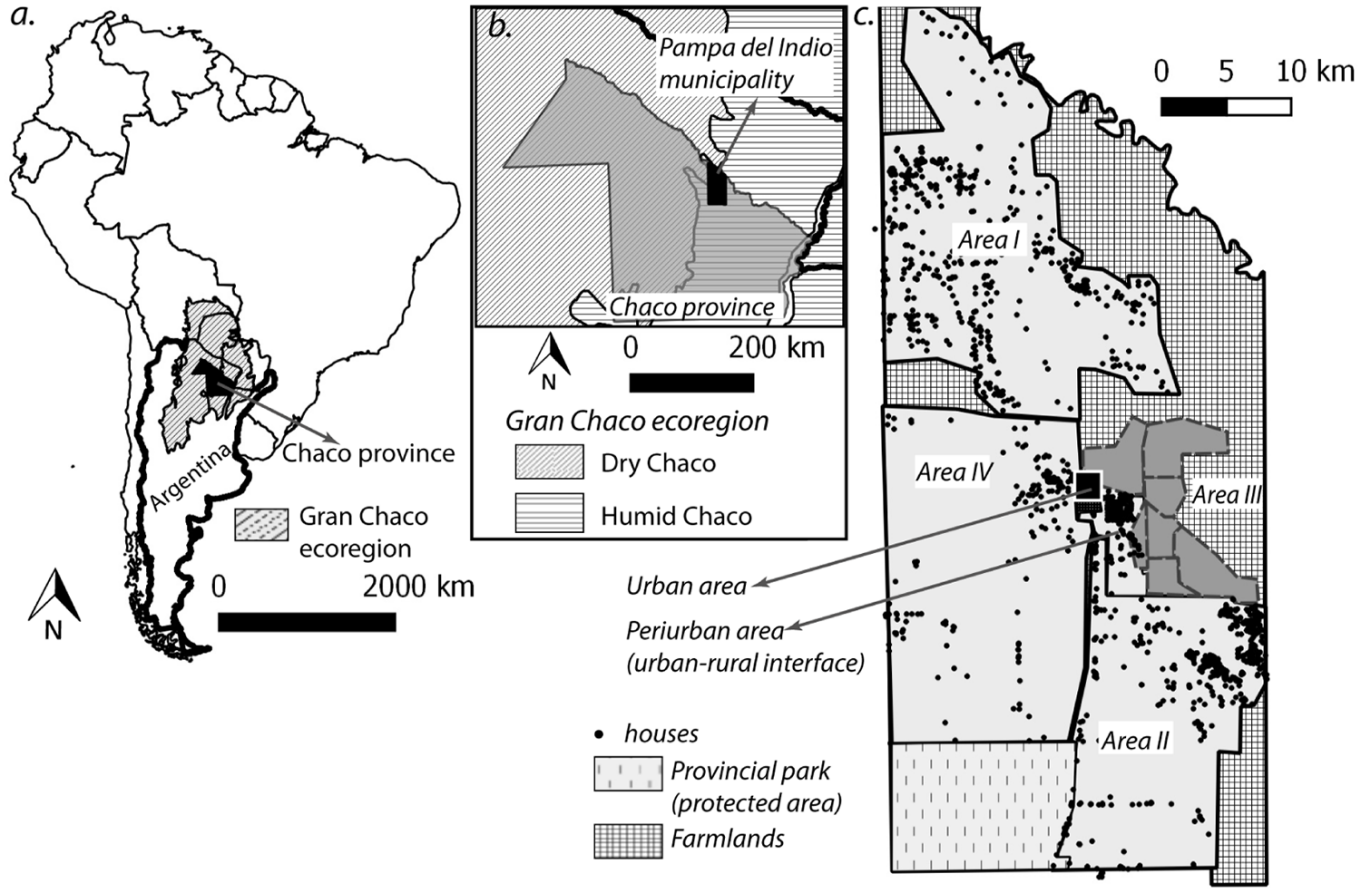
Map of the study area. a. Location of Chaco Province, northeastern Argentina, within the Gran Chaco ecoregion. b. Location of Pampa del Indio municipality within Chaco Province. c. Urban, peri-urban and rural areas (I-IV) of the municipality.

Fieldwork was conducted in a 95 km^2^ rural section of Pampa del Indio municipality (25° 55’ S 56° 58’ W), Chaco province, Argentina, denominated Area III. The rural area of the municipality was divided in four sections (I-IV) in which a similar intervention protocol was performed (Fig 1). Area III included 404 occupied (inhabited) house compounds in seven communities where Qom households predominated over a Creole minority as of October 2008 (baseline). The main features of the study area and its population were described elsewhere [40]. The study houses were surrounded by agricultural fields mixed with patches of native forest subject to variable degrees of degradation. No significant environmental changes were observed in the study communities during the follow-up. Official census information at the municipality scale indicated a very large annual population growth rate (4.9%) over the 2001–2010 period. An average Qom household had 6.4 occupants in a 43.4-m^2^ sleeping quarter (domicile), whereas local Creoles had 4.1 occupants in a nearly twice as large area. Houses lacked access to safe water and a sewage or garbage disposal system within their premises [40].

The last insecticide spraying campaign targeting house infestation with *T*. *infestans* in Pampa del Indio municipality had taken place in 1997–1998. Additionally, a few clusters of Area III houses were selectively sprayed by local healthcare personnel in 2000 (36 houses), 2006 (49 houses), and July 2008 (45 houses from two communities). Although these actions were not recorded by the provincial Chagas control program, local householders’ reports to the research team agreed with the information provided by local healthcare agents [40].

### Study design

A longitudinal study was conducted to monitor house infestation with triatomines before and after community-wide spraying with insecticides (Table 1) and any parallel change in housing structure and household composition. Our usage of household- and housing-related terms follow definitions appearing elsewhere [47,48]. The condition of each house unit was registered at each vector survey and classified as follows: i) occupied: inhabited; ii) vacant: uninhabited, with clear signs of current vacancy, often corroborated by neighbors; iii) closed: showing signs of being occupied, often confirmed by neighbors, with residents absent after one to three re-visits; iv) demolished: no longer existing at its exact previous location, and v) new house unit: no previous record at its exact current location. A household was defined as the people (related or not) sharing food and other housekeeping activities despite the fact that sometimes there were two or more structurally separate sleeping quarters occupied by related family (i.e., extended family). In our specific context one household equaled one house unit. A house compound consists of one or more separate human sleeping quarters (domicile or domestic premises), a patio and nearby peridomestic buildings for human use and animals (e.g., kitchen, storeroom and chicken coop, among others) as illustrated elsewhere [40,49]. The study protocol was approved by the Dr. Carlos A. Barclay Independent Ethical Committee for Clinical Research, Buenos Aires, Argentina (IRB No. 00001678; Protocol N ° TW-01-004, Revision N° 863-32-2011).

**Table 1:** Type and coverage of house infestation surveys and spraying with pyrethroid insecticides in Area III of Pampa del Indio, 2008–2015. Only occupied houses are included.

Months post-spraying (Month/year)	Intended vector survey coverage	Type of spraying	Pyrethroid dose^a^	% spray coverage (No. houses sprayed)
0 (10/2008)	Total	Community-wide	Simple	96.0 (404)^b^
10 (8/2009)	Total	Selective	Simple	100.0 (46)^c^
18 (4/2010)	Total	Selective	Simple	100.0 (2)
38 (12/2011)	Partial	Selective	Double	100.0 (2)
49 (11/2012)	Total	Selective	Simple	100.0 (3)
59 (9/2013)	Partial	Selective	Simple	0.0 (0)
78 (4/2015)	Total	Selective	Simple	0.0 (0)

^a^ Suspension concentrate beta-cypermethrin (50 mg/m^2^) or deltamethrin (25 mg/m^2^) for community-wide spraying; deltamethrin was applied during the vector surveillance phase except in 2009 (10 MPS) when beta-cypermethrin was used (the only insecticide available at the vector control program).

^b^ Includes 26 houses sprayed by local healthcare agents in July 2008 and not re-sprayed five months later when the community-wide insecticide campaign was undertaken.

^c^ All infested and newly-built houses (not sprayed during the insecticidal campaign) were sprayed with insecticides.

During our first visit to the area, we explained the purpose of the study to the heads of each household invited them to participate and asked them for an oral consent. A small aluminum plate was nailed to the front door to identify each house unit for detailed follow-up. The geographic location of each house compound was recorded with a GPS (Garmin Legend) and maps were generated with QGIS 2.18.16. The geographical coordinates of each house compound were transformed to preserve the privacy of the households involved in this study, as described elsewhere [40].

### Vector surveys

All house compounds were inspected for triatomines by timed-manual searches using a dislodjant aerosol (0.2% tetramethrin) (Espacial, Argentina) before (October 2008) and periodically after community-wide insecticide spraying (Table 1): August 2009 (10 MPS), April 2010 (18 MPS), November 2012 (49 MPS) and April 2015 (78 MPS). Each domicile and peridomestic structure at each compound was searched by one person for 15 min; searches and insecticide applications were performed by skilled personnel from the national or provincial vector control programs [40]. One member of the research team was present in almost every inspected or sprayed house during the entire follow-up. Closed houses were re-visited at least once, and sometimes two or three times, to achieve full-inspection coverage at each survey. Public buildings and vacant houses were inspected for triatomine infestation when they were occasionally used as a dwelling or as resting places by free-ranging domestic animals, respectively; because none of them were ever found infested, they were excluded from house infestation estimates. *T*. *infestans* was found by timed-manual searches in 28.0% of the occupied houses inspected at baseline, and in 31.9% of them when all vector detection methods were considered [40]. Triatomine abundance was calculated as the number of bugs collected by timed-manual searches per unit of catch effort. Colonization was defined as the presence of any nymphal instar among the triatomines collected.

The community-wide insecticide spraying campaign covered 96.0% of all occupied houses in October 2008 (0 MPS) (Table 1). Houses infested with *T*. *infestans* over 2009–2015 were selectively sprayed with pyrethroid insecticide immediately after each survey.

In order to promote community-based vector surveillance, householders were asked for the presence of triatomines in their dwellings and shown dry specimens of the local species at every vector survey, and were instructed to collect any triatomine they may find and bring it to the local healthcare post or hospital. This information was used to build two house infestation indices: householders’ bug collection (when they delivered triatomines) and householders’ bug notification (when only reports were given). In October 2011 (36 MPS) and September 2013 (59 MPS), local healthcare agents aimed at visiting every household to ask residents for the presence of triatomines in their dwellings; no dry triatomine specimens were shown. Research team members surveyed additional households on December 2011 (38 MPS) to increase the coverage of residents’ notifications; in total, 85% of all occupied house units (373) were surveyed over October-December 2011. During these visits, timed-manual searches were performed in a selected sample of the occupied houses that either reported the presence of triatomines and/or had a previous infestation. Additionally, a random sample of dwellings with suitable conditions for infestation (i.e., mud or unplastered walls, tarred-cardboard roofs, high refuge availability, large household size, as defined below) were also inspected by timed searches. In September 2013 (59 MPS), 200 houses (48% of all occupied house units) were visited by local healthcare workers to canvas householders on house infestation; timed-manual searches were later performed following the same criteria described above.

All triatomines collected during the follow-up were carried to the field laboratory for identification to species, stage and sex, and then were preserved at -20°C. Feces of all live third-instar nymphs and later stages were examined for infection with *T*. *cruzi* at 400× within 2–4 weeks of bug collection as described elsewhere [50].

### Socio-demographic and environmental surveys

Detailed socio-demographic and environmental surveys were performed in parallel to vector surveys in October 2008 (0 MPS), November 2012 (49 MPS) and April 2015 (78 MPS). At each occupied house, an adult household member fluent in Spanish was asked for information on demographic (i.e., number of residents by age class and gender), economic (i.e., number and type of domestic animals, and their resting places) and environmental variables (e.g, construction features, insecticide use) to monitor changes over time and investigate their association with house infestation [40]. For each domicile, the building materials used in roof and walls, presence and type of wall plaster, condition of wall surface, type of floor, and number of sleeping quarters were recorded in a form. The availability of refuges for triatomines was categorized into five levels by a skilled member of the research team [49]. Additional socio-demographic variables were registered from 49 MPS on, including land ownership, educational level of each household member, and whether a new domicile or house was provided by a government-sponsored rural housing program or not. These data were used to compute household educational level, defined as the mean number of schooling years attained by household members aged 15 years old or more, and the overcrowding index, defined as the number of human occupants per sleeping quarter [40].

Housing improvement referred to houses with mud-walled domiciles (or more rarely, built with other materials such as wood or plastic) that shifted to having one or more new human sleeping quarters with brick-and-cement walls and a corrugated metal-sheet roof (the only type of improved roof recorded, frequently denominated modern houses), regardless of whether they had been provided by the rural housing program, house residents or any third party. These changes were assessed in relation to the status at the preceding survey in which housing-related variables were registered (0, 49 and 78 MPS surveys). When more than one building material or more than one domicile existed at baseline, the maximum quality level qualified the status of the house unit. For example, if a house had one mud-walled domicile and a second one with brick-and-cement walls, the house unit was taken to have brick-and-cement walls. Only stable houses provided the data needed for this analysis (see definitions below).

The demographic composition and location of each household, and the residential destination of those who moved elsewhere between surveys and their main reasons, were recorded at 49 and 78 MPS. These data were used to classify household mobility for the entire follow-up (0–78 MPS) and for each study period (0–49 MPS and 49–78 MPS), and varied slightly from the classification we used previously [40]: movers were households that changed its exact residential location within Area III (i.e., local movers); non-movers were households that remained at the exact residential location; out-migrants were households that changed its residential location to (peri-)urban sections of the municipality or other cities within the country; new households, those established in Area III during the follow-up (owing to the formation of a new family or by in-migration, regardless of their origin), and households that ceased to exist (owing to separation, demise of the only resident, or permanent out-migration from Area III).

Housing stability between any two surveys (0, 49 and 78 MPS) was classified in three levels: stable (i.e., a permanently occupied house unit), non-stable (a demolished or vacant house unit), and new (i.e., a newly-built house unit, regardless of the source, type or state of building materials).

### Pyrethroid-resistance bioassays

A sample of the *T*. *infestans* collected at baseline (64 females from 22 (18%) house units in 5 of the 7 infested communities) was tested for pyrethroid resistance at the Center for Research on Plagues and Insecticides (CIPEIN/CONICET, Buenos Aires, Argentina) using standardized methods [51]. Screening coverage ranged between 13% and 25% of the infested houses across communities.

The screening bioassays consisted in the application of a discriminating dose (DD) of 2 ng of technical-grade deltamethrin (99%), provided by Ehrenstorfer (Augsburg, Germany) per insect, which causes 99% mortality of the susceptible strain (DL99) [52]. First-instar nymphs of *T*. *infestans* (5–7 days old, mean weight 1.3 ± 0.2 mg, unfed) (F1) received a topical application of 0.2 μl of deltamethrin (0.01 mg/ml) in analytical grade acetone (Merck, Buenos Aires, Argentina) on the dorsal abdomen using a 10-μl Hamilton syringe (Hamilton PB-600-1, Nevada, USA) with an automatic repeating dispenser [51,79]. The treated insects were kept inside a plastic container with folded paper at 28–30 °C and 50–70% RH. Mortality was evaluated after 24 h by placing the insects at the center of a circular filter paper of 11 cm diameter; nymphs able to walk to the border were taken as survivors [51]. The bioassays consisted of three replicates containing at least 10 insects for each bug population. A laboratory-reared, deltamethrin-susceptible colony of *T*. *infestans* (denominated CIPEIN SRL, susceptible reference lineage), was used as a negative control, and a laboratory-reared pyrethroid-resistant *T*. *infestans* colony (from Salta, Argentina) was used as a positive control.

A triatomine population was considered resistant to the insecticide tested if mortality was less than 90% in two out of three independent assays, i.e., at least one survivor in two of 3 trials [52]. When the number of first-instar nymphs available only allowed one or two independent assays to be performed, the outcome was taken to be resistant or susceptible pending confirmation. Houses were grouped according to median mortality in the bioassays as follows: susceptible (91–100%), moderate (76–90%), reduced (50–75%). No sample showed mortality rates fewer than 50%.

### Data analysis

The percentage of new, occupied house units at the survey conducted at time *t* was calculated relative to the total number of occupied house units enumerated at *t*. The percentage of demolished houses at *t* was computed from the number of occupied houses enumerated at *t-1* that ceased to exist at *t* relative to the number of occupied houses enumerated at *t-1*.

All statistical analyses were conducted using Stata 15.1 [53]. The distribution of household characteristics over the follow-up was examined with χ^*2*^ tests. The Kruskal-Wallis test was used for comparison of household size over time. The agreement between householders’ notifications of triatomine presence and direct assessments of house infestation was measured using the kappa index. An exact Fisher’s test was used to examine the association between house infestation status before and after insecticide spraying. A Cox regression survival analysis was used to test whether the loss rate of house occupancy differed between infested and non-infested houses at baseline.

Global spatial analyses (univariate and bivariate) were performed using the weighted K-function implemented in Programita [54]. Random labeling was selected to test the null hypothesis of random occurrence of events among the fixed spatial distribution of all houses. The selected cell size was 200 m (assuming that each house had at least three neighbors at the minimum distance of analysis), and the maximum distance was set at 6 km (i.e., half of the dimension of the area) [55]. Monte Carlo simulations (n = 999) were performed and the 95% ‘confidence envelope’ was calculated with the 2.5% upper and lower simulations. We created heat maps (i.e. density maps) to visualize the spatial aggregation of housing instability (i.e., non-stable houses) over 0–78 MPS. The analysis was implemented in QGIS using a kernel density estimation algorithm within a radius of 200 m.

## Results

### Household and housing follow-up

**Fig 2:**
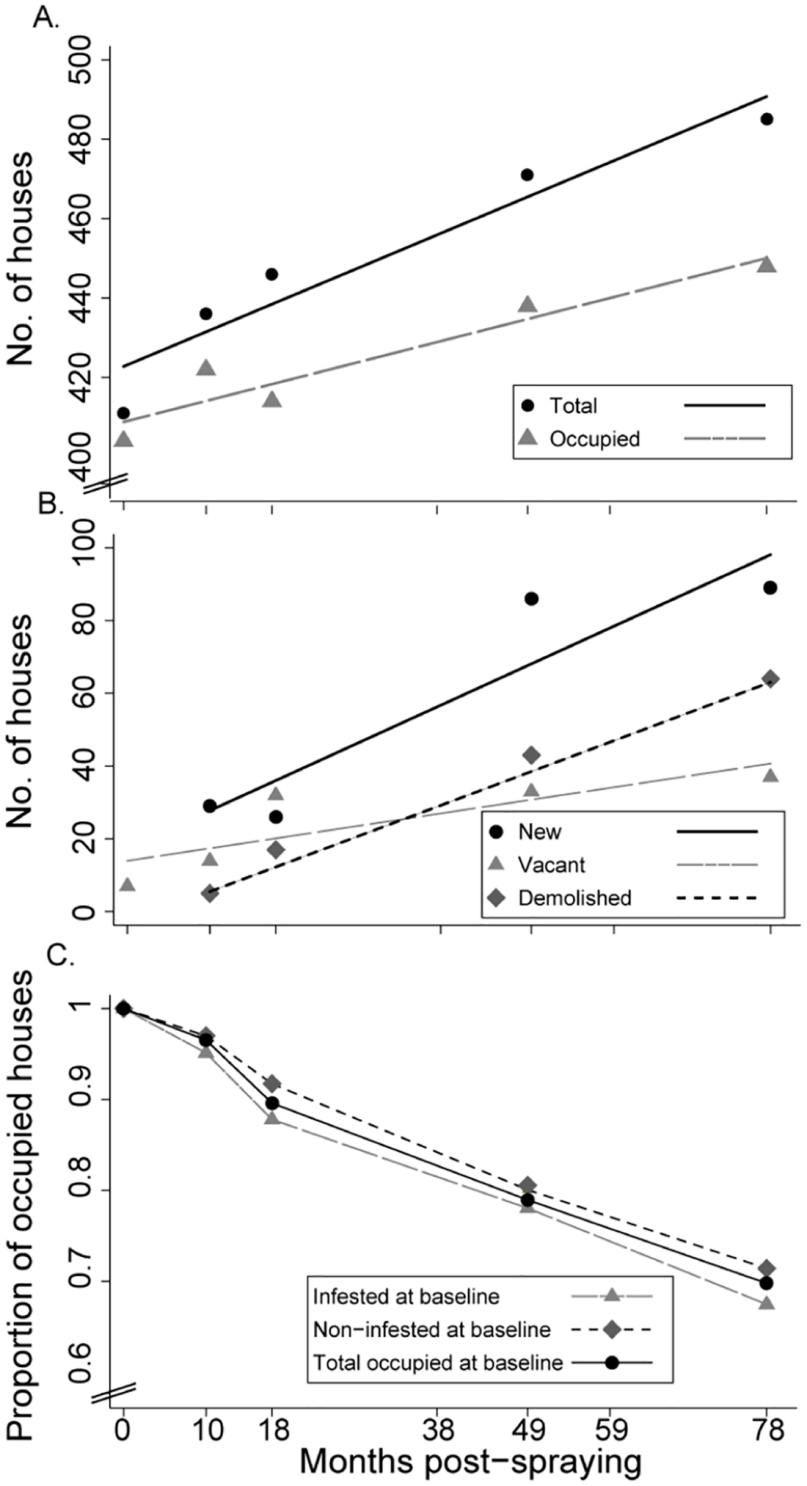
Status of house units over time (A: total and occupied; B: new, demolished and vacant) and survival curves of baseline-infested, -non-infested and -occupied house units (C) in Area III of Pampa del Indio, 2008–2015.

The total number of houses increased from 411 to 485 in a roughly linear fashion over the seven-year period, as did the frequency of occupied, new and demolished houses (Fig 2A and 2B). The observed frequencies and linear regression equations appear in S1 Table. In total, 30.7% of the occupied houses at baseline were lost (i.e., vacant or demolished) over the follow-up; the annual loss rate was 4.6% (standard error, 0.36) as determined by ordinary linear regression (F = 168.1, df = 1 and 3, p < 0.001, adj R^2^ = 0.977). Among the 123 baseline-infested houses (as determined by any collection method), 22.0% and 32.5% subsequently became vacant or were demolished at 49 and 78 MPS, respectively, whereas 19.9% and 28.6% of the 266 non-infested houses at baseline were no longer occupied at those time points, respectively (Fig 2C) (Cox regression test, χ^*2*^ = 0.2, df = 1, p = 0.7).

The prevalence of house units having at least one domicile with mud walls decreased marginally from 78.9% to 72.4% over 0–78 MPS (χ^*2*^ = 4.9, df = 2, p = 0.09), whereas the same metric for brick-and-cement walls increased highly significantly from 25.8% to 41.9% (χ^*2*^ = 24.5, df = 2, p < 0.001) (Table 2). These changes were closely related to a government-sponsored rural housing program, which increasingly covered up to 18.6% of existing houses as of 78 MPS and benefited 77 Qom and two creole households. This increase in the proportion of brick-and-cement walled domiciles was even observed in the group of baseline-infested houses (S1 Text). Nonetheless, most of the new house units (236) built during the follow-up had unplastered mud-walled domiciles (S1 Text). For all houses with an improved domicile at 78 MPS, most of the new premises had plastered walls (73.3%) and absence of cracks (81.7%), whereas 66–94% of them also retained the former mud-walled structure at 49 and 78 MPS. Almost 60% of all mud-walled houses had unplastered walls as of 78 MPS.

**Table 2:** Characteristics of domestic premises, household size and vector control practices in Area III of Pampa del Indio, 2008–2015.

Attribute	% of houses (No. of houses surveyed)		
	0 MPS	49 MPS	78 MPS
Mud walls	78.9 (399)	75.1 (438)	72.4 (445)
Brick-and-cement walls	25.8 (399)	33.3 (438)	42.0 (445)
Tarred-cardboard roofs	52.9 (399)	38.4 (409)	31.6 (433)
Rural housing program^a^	NR*	8.3 (423)	18.6 (427)
Refuge availability (mean ± SD)	3.8 ± 0.8	4.1 ± 0.8	4.0 ± 0.7
Domestic insecticide application	47.7 (386)	65.7 (411)	NR*
Household size (mean ± SD)	6.2 ± 3.5	5.8 ± 3.4	5.9 ± 3.5
Residential overcrowding^b^ (mean ± SD)	NR*	3.5 ± 2.3	3.4 ± 2.1
Household educational level^c^ (mean ± SD)	NR*	5.3 ± 3.1	5.1 ± 3.1

^a^ Fraction of all surveyed house units built by the housing program, which included human sleeping quarters with brick-and-cement walls, corrugated metal-sheet roof and cement floor.

^b^ Number of residents per sleeping quarter.

^c^ Mean number of years of schooling among residents aged ˃15 years old.

*Data not registered (NR).

The trend toward housing improvement was also expressed in the highly significant drop in houses having tarred-cardboard roofs, from 52.9% to 31.6% over 0–78 MPS (χ^*2*^ = 40.5, df = 2, p < 0.001), which were replaced by corrugated metal-sheet roofs (Table 2). In spite of these improvements, the mean score of domestic refuge availability for triatomines remained high and tended to increase (Kruskal-Wallis test, χ^*2*^ = 6.8, df = 2, p = 0.03). Householders’ application of domestic insecticides mainly included low-concentration pyrethroid sprays; their frequency of use highly significantly increased over 0–49 MPS (χ^*2*^ = 26.4, df = 1, p < 0.001). Household size averaged 6 residents and remained approximately constant (Kruskal-Wallis test, χ^*2*^ = 4.7, df = 2, p = 0.09), as did residential overcrowding and household educational level.

Most households (>78.5%) had stable residence over 0–49 and 49–78 MPS (Table 3). Movements mainly occurred within the study area (i.e., local movers, ~11%), and slightly less often included out-migration to large cities or (peri-)urban areas of Pampa del Indio (7.5–9.2%). The annual rate of household mobility averaged 5.1% and 8.0% over 0–49 and 49–78 MPS, respectively. Most movers comprised Qom households (96.0%, 95/99), who torn down (77.8%) their former dwelling and occupied a new house (98.3%) rather than an existing one. Significantly fewer (54.9%, 39/71) out-migrant households demolished their houses compared to movers (χ^*2*^ = 10.0, df = 1, p = 0.002). Nearly half (45.3–51.0%) of the new houses were occupied by movers (Table 3). The formation of new households from current Area III residents and from in-migrants substantially increased (by 3.9–11.6×) over 49–78 MPS relative to 0–49 MPS. Most in-migrants (86%, 18/21) occupied a new house. Overall, 47% of the households that changed their residential location over 49–78 MPS had also moved or migrated at least once over 0–49 MPS (repeat movers).

**Table 3:** Distribution of household mobility and origin of new occupants in Area III of Pampa del Indio, 2008–2012 and 2012–2015.

Variable	Level	% (no. of house units)	
		0–49 MPS	49–78 MPS
Household mobility*	Non-movers	78.5 (317)	79.8 (352)
	Movers	11.6 (47)	11.8 (52)
	Out-migrants^1^	9.2 (37)	7.5 (33)
	Other^2^	0.7 (3)	0.9 (4)
Origin of occupants of new houses	Movers	51.0 (76)	45.3 (39)
	New households	2.7 (4)	31.4 (27)
	In-migration	6.0 (9)	23.3 (20)
	No data	40.3 (60)	- (0)

* Only includes stable and non-stable housing units.

^1^ Includes permanent movement to large cities or (peri-) urban areas of Pampa del Indio, and absence of an exact destination.

^2^ The only dweller passed away.

### House infestation and triatomine infection

**Fig 3:**
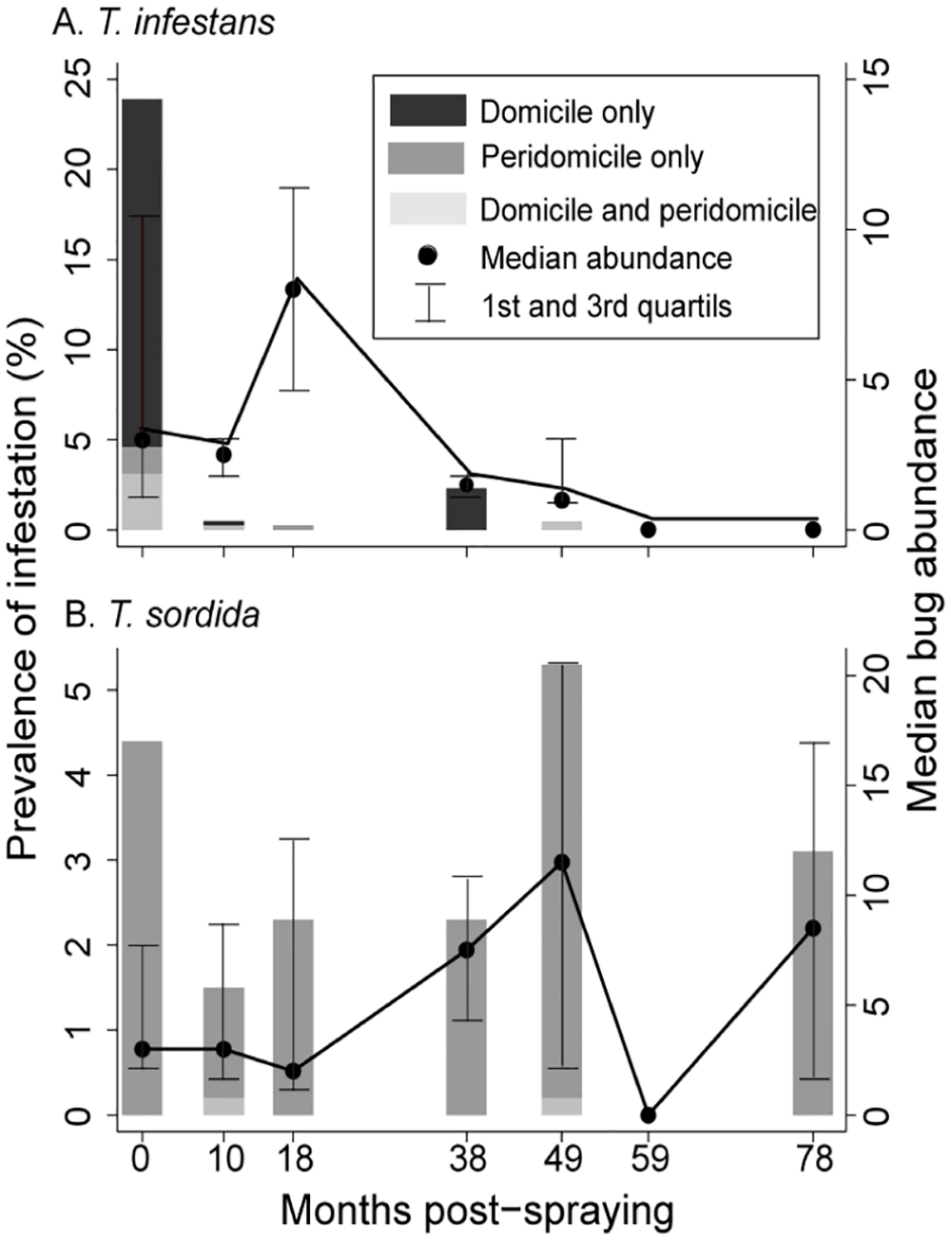
Prevalence of house infestation (bars) and relative abundance (circles: median; bars: first and third quartiles) of A) *Triatoma infestans* and B) *Triatoma sordida* in domestic and peridomestic premises in Area III of Pampa del Indio, 2008–2015. Numbers above bars indicate the number of occupied houses inspected in each survey. The y axis scale differs between graphs.

House infestation decreased from 31.9% to 0.7% at 10 MPS and then remained marginal, with no infested house detected at 59 and 78 MPS (Fig 3A). Vector surveys inspected 93.8–96.3% of the occupied house units over the follow-up; less than 1.6% of them refused searches for triatomines (S1 Table). No public building or vacant house was ever found infested with *T*. *infestans*. The few occupied houses that were neither inspected for infestation nor sprayed with insecticides at baseline were subsequently inspected for triatomines at least once, and none of them was positive for *T*. *infestans* except one at 10 MPS (which had neither been inspected nor sprayed at baseline due to its remote location). The apparent increase in house infestation (2.3%, only including two infested houses) at 38 MPS was related to targeted searches of higher-risk dwellings.

Pre- and post-spraying infestation mostly occurred in human sleeping quarters. Only 10 (1.6%) of 615 occupied houses inspected at least once over the follow-up were positive for *T*. *infestans* by timed-manual searches, and 9 of them had infested domestic premises. The median relative abundance per infested collection site over the follow-up was low (2.0 triatomines; first-third quartiles, 1–3 per unit of catch effort), and peaked at 18 MPS owing to a high-density triatomine population in a peridomestic habitat used by chickens (Fig 3A). Colonies of *T*. *infestans* were found in 8 of the 10 infested houses as determined by any detection method. The stages most frequently captured by timed-manual searches were females (33%) and males (17%).

*Triatoma sordida* (a secondary vector candidate for domestication) was found by timed-manual searches in 4.4% of the occupied houses at baseline, and then fluctuated between 1.7% and 5.3% over the follow-up (excluding surveys in which only a sample of houses was inspected) (Fig 3B). *T*. *sordida* mainly occurred in habitats used by chickens, and was collected inside a human sleeping quarter or a kitchen (both adults and nymphs) only in two occasions. The median relative abundance per infested collection site was 7 triatomines (first-third quartiles, 1–14 per unit of catch effort) over the follow-up; colonies were found in 90.2% of all infested sites as determined by any method. Fifth- (32%) and fourth-instar nymphs (23%) were the stages most frequently captured by timed searches.

Baseline and post-spraying house infestation with *T*. *infestans* were not significantly associated among 379 houses inspected for triatomines on both periods (exact Fisher’s text, p = 0.683, df = 1) (S2 Table). Three of the post-spraying infested houses had also been infested at baseline, but in between these occasions there were 1 or 2 surveys in which no *T*. *infestans* was collected by any method, suggesting post-spraying infestations were unlikely to be residual foci. The three infested houses at 10 MPS (two baseline-negative and one baseline-no data) were not strictly defined residual foci because they had not been sprayed with insecticides at baseline, although two of them might have been residual foci from a prior non-professional spraying. The remainder was neither inspected nor sprayed at baseline due to its remote location (footnotes 3 and 4 in S2 Table).

The space-time series of observations and subsequent interviews to householders suggested putative cases of passive or active dispersal of *T*. *infestans* among infested dwellings of one community (Pampa Grande). Four of the five infested houses detected at 38 and 49 MPS were located in a well-defined sector in which only one house was heavily infested (i.e., the putative index case). Householders reported that one resident of the heavily infested house moved temporally with his belongings to two nearby houses, which subsequently appeared infested, suggesting passive bug transport. The other infested house was at 700 m of the heavily infested one, and within the flight range of *T*. *infestans*.

Three of the 10 infested house units detected over the follow-up harbored at least one *T*. *infestans* infected with *T*. *cruzi* whereas this fraction (34/75, including only infested houses with bug infection data) was higher at baseline though not significantly so (Fisher’s exact test, p = 0.5). This downward trend holds both in domiciles (33%, 3/9 vs. 45%, 28/62) and peridomiciles (0%, 0/3 vs. 44%, 8/18), respectively. The overall infection prevalence in post-spraying *T*. *infestans* was 11% (n = 44 insects examined). None of the 42 *T*. *sordida* collected from 6 houses at 10 MPS were infected with *T*. *cruzi*.

### Community-based vector surveillance

**Fig 4:**
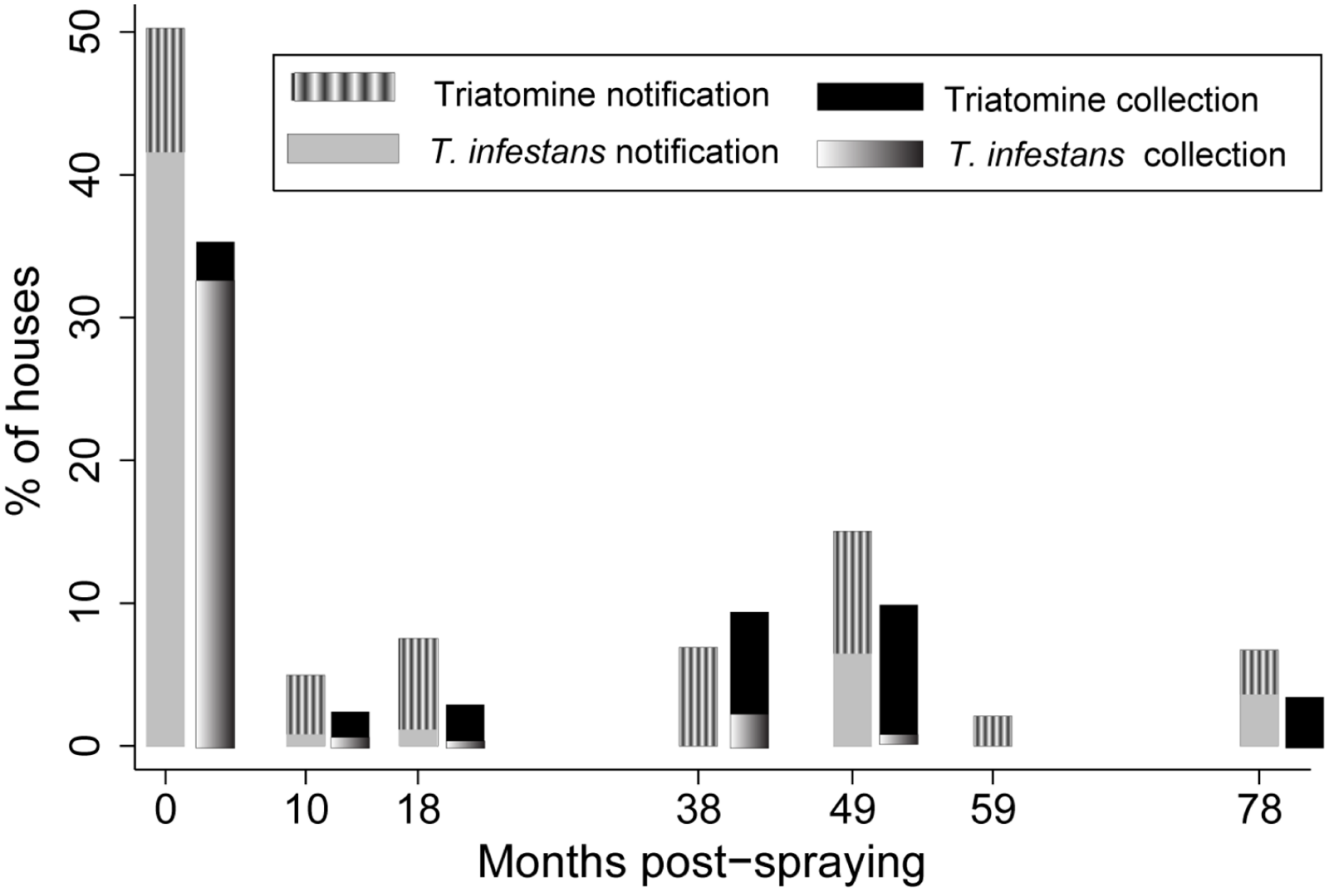
Comparison between householders’ notifications of the presence of *T*. *infestans* or any triatomine species and the outcome of other vector detection methods (including timed-manual searches, during insecticide spraying and householders’ bug collections) in Area III of Pampa del Indio, 2008–2015. Only notifications of Triatominae (without distinguishing species) were registered at 38 and 59 MPS.

Householders’ notifications of the presence of any triatomine species were significantly more frequent than the direct assessments by any detection method at every post-spraying survey (χ^2^ test, df = 1, p < 0.01), except at 10 MPS (χ^2^ test, df = 1, p = 0.4) (Fig 4). There was a poor agreement between post-spraying householders’ notifications and other vector detection methods (kappa coefficients < 0.2). Nonetheless, the relative odds of post-spraying house infestation with *T*. *infestans* was approximately 10–13 times higher when householders notified the presence of any triatomine than when they did not (10 MPS: OR, 10.1; 95% CI: 0.9–118.5; 18 MPS: OR, 12.6; 95% CI: 0.7–211.9, and 49 MPS: OR, 11.7; 95% CI: 1.03–134.3). Householders’ notification of *T*. *infestans* also dropped below 1% at 10 MPS, as did timed-manual searches, and then increased at 49 MPS (6.5%) and 78 MPS (3.6%) (Fig 4), matching the frequent catch of *T*. *sordida* by timed searches in both surveys (Fig 3B) and householders’ collections of *T*. *sordida* or other triatomines at 49 MPS. Householders reported the presence of *T*. *infestans* before timed searches in five of the 10 houses ever found infested post-spraying; the remainder had either a peridomestic infestation only or very low domestic bug abundance. Householders collected 45 triatomines at 25 different dwellings and other Reduviidae bugs at 15 houses over the surveillance phase (S1 Text).

### Insecticide resistance and house infestation

In total, 55% of the 22 populations of *T*. *infestans* individually screened for pyrethroid resistance had reduced mortality (Table 4). None of the post-spraying infested houses had information on pyrethroid resistance at baseline. Moreover, none of the 12 houses with reduced mortality to pyrethroids was found infested after the community-wide spraying.

**Table 4:** Mortality of *T*. *infestans* populations in pyrethroid-resistance bioassays, Area III of Pampa del Indio, 2008 (baseline).

Deltamethrin resistance status (% mortality)	No. of houses	% median mortality (first-third quartiles)
Susceptible (91–100%)	10	100
Moderate (76–90%)	8	83.5 (80.8–90.0)
Reduced (50–75%)	4	58.0 (56.0–68.8)

### Housing instability, improvement and house infestation

**Fig 5:**
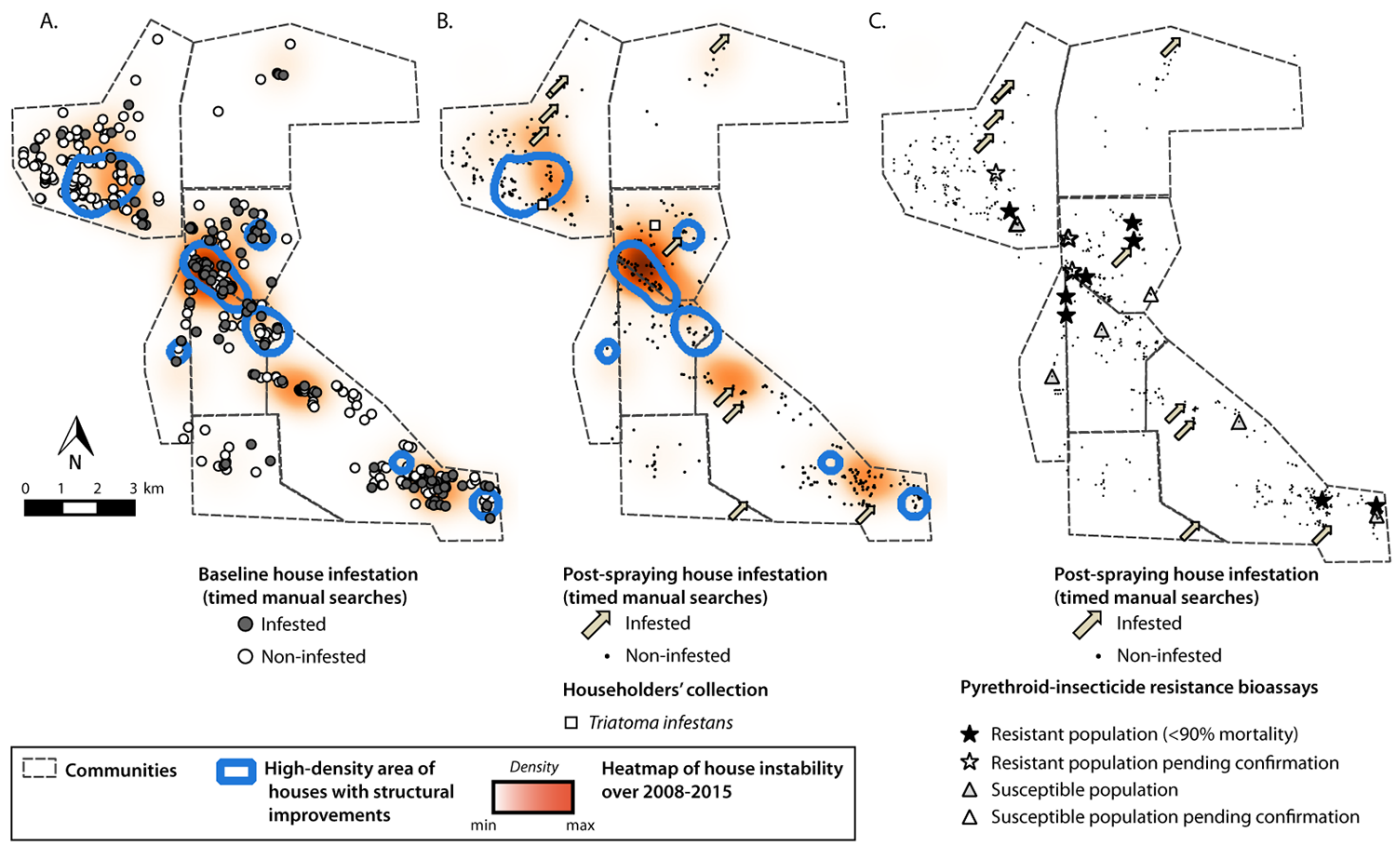
**Spatial distribution of baseline (A) and post-spraying (B) house infestation with *T*. *infestans* (circles), housing instability (heat map) and high-density areas with housing improvements (contour lines). C. Evidence of pyrethroid resistance at baseline and post-spraying house infestation with *T*. *infestans* in Area III of Pampa del Indio, 2008–2015.** The maps were created in QGIS 2.18.16. based on the data collected within the scope of this study (S3 Table).

Housing instability was mainly concentrated in a few clusters of houses, as was housing improvements. Both events partially overlapped, particularly in a section with high-density residential mobility and housing improvement (Fig 5A and 5B). The spatial distribution of non-stable houses and of improved houses did not differ significantly from a random pattern. However, a qualitative spatial overlap between a high density of improved houses, housing instability and baseline house infestation is apparent (Fig 5A), and wanes for post-spraying house infestation (Fig 5B). No significant association was found between post-spraying house infestation and household instability or housing improvements in univariate analyses (χ^2^ = 0.5, df = 1, p = 0.5; χ^2^ = 0.8, df = 1, p = 0.4, respectively). Both the houses with any evidence of pyrethroid resistance and those screened for the latter were dispersed throughout the study area (Fig 5C), showing no apparent spatial association with post-spraying infestation.

The relationship between post-spraying domestic infestation with *T*. *infestans*, housing stability and housing improvement is shown in Table 5. Non-stable and stable houses displayed virtually the same prevalence of domestic infestation, which was twice as large as that of new houses. None of the 29 houses subjected to improvement became infested. When differential exposure time was accounted for, non-stable, non-improved houses had a much higher rate of domestic infestation than stable, non-improved houses (~×4) or new houses (~×2). This ranking was not affected by whether the three putative residual foci were excluded or not (footnote of Table 5).

**Table 5:** Post-spraying domestic infestation with *Triatoma infestans* according to housing stability and improvements in Area III of Pampa del Indio, 2008–2012. The table includes only occupied houses inspected at least once over 10–49 MPS and excludes the three putative residual foci detected at 10 MPS.

Housing stability*	Housing improvement**	Prevalence of domestic infestation (no. houses infested/ no. inspected)	Rate of post-spraying domestic infestation per 100 house-years (exposure time)
Non-stable houses	No	1.5 (1/67)	1.1 (90.2)
Stable houses	No	1.4 (4/288)***	0.3 (1176.0)***
	Yes	0.0 (0/29)	0.0 (118.4)
New houses	-	0.7 (1/141)	0.6 (159.2)

* ‘Non-stable’ includes occupied houses at baseline that subsequent became vacant or were demolished over the follow-up; ‘Stable’ includes houses that were permanently occupied over the follow-up; ‘New houses’ include occupied houses that were built after the baseline survey.

** Housing improvement refers to a house that shifted from a mud-walled domicile (or wood or plastic) to one having brick-and-cement walls and a corrugated metal-sheet roof.

*** If the three putative residual foci detected at 10 MPS were included, the prevalence of domestic infestation reaches 2.4% (7/291) and the rate 0.6% (over 1188.3 house-years).

## Discussion

Our seven-year intervention program exerted immediate impacts on *T*. *infestans* populations and infected-bug densities, which were sustained at least through 78 MPS in a high-risk, remote setting including vulnerable indigenous communities despite evidence of moderate pyrethroid resistance in the main vector. These results refute the assumption that vector control actions performed in marginalized rural communities of the Gran Chaco are doomed to fail and lead to rapid house reinfestation (e.g., [10, 36, 37, 56]). The prevalence of post-spraying house infestation, vector abundance and infection rates in Area III were substantially lower than in neighboring rural areas subjected to the same intervention protocol [11,38] or in other rural locations within the Gran Chaco [10, 36, 56–59]. The larger impacts of the interventions in Area III were likely associated with the combined effects of high-coverage, professional insecticide spraying and systematic vector surveillance-and-response, frequent housing replacement and residential mobility, and broad geographic coverage of sustained vector control creating a buffer zone, since Area III was surrounded by virtually uninfested communities and agricultural fields (Fig 1).

Other ecological factors may have contributed to the greater relative impact of vector control actions in Area III: i) pre-spraying house infestation mainly occurred inside domestic premises, where the effectiveness of pyrethroids and duration of its residual effects are much higher than in peridomestic structures exposed to weather agents [11, 56, 57, 60]; ii) houses had fewer or no peridomestic structures with hosts (i.e., fewer suitable habitats for triatomines); iii) stochastic processes affecting the recovery of low-density, isolated triatomine populations are likely to occur, and iv) the study communities had fewer forest fragments near dwellings, which may eventually harbor sylvatic foci of *T*. *infestans*, not detected in Pampa del Indio yet [61]. This may not represent a barrier to house invasion with triatomines. Henceforth we examine the main outcomes in terms of structural determinants (housing quality dynamics), demographic features (household mobility), and vector-related aspects.

Inadequate and unhealthy housing prevailed over time and space in the study area. Mud-walled houses were similarly prevalent at baseline and among the newly-built houses, whereas the fraction with brick-and-cement walls and metal roofs increased slowly over time, partially through a government-sponsored housing program. The weak trend toward housing improvement did not translate into an overall reduction of unplastered mud walls (nor in the mean score of domestic refuge availability) because most households kept the former structure owing to its greater capacity to dampen extreme temperatures or for alternative usage, and because most of the new buildings were mud-walled. Rather than replacement of precarious houses, there was a disproportionate addition of precarious to modern housing units (~4:1). Other improvements (in peridomestic structures and screening of doors and windows) were not implemented except for the latrines built by the housing program. In other settings, the frequent use of corrugated metal roofs lacking an insulating ceiling underneath created an unbearably hot, unhealthy environment that defeats the goal of replacing inadequate dwellings. Thus, housing programs must consider the environmental and cultural features of the target populations and foster community participation in the design, layout and arrangement of house compounds to achieve the purported goals.

Even higher-quality modern houses can be heavily infested with *T*. *infestans*, as there are numerous refuges for triatomines in boxes and furniture regardless of other structural aspects of housing quality, and triatomines may hide in the insulating ceiling (S2 Fig). Although housing improvements may reduce refuge availability, triatomine abundance and domestic transmission risks [31–34, 36, 62, 63], and provide another means to cope with pyrethroid-resistant triatomines [64], the effects may range from subtle to strong depending on the details, scale and context of interventions. For example, the massive rural housing program implemented in Venezuela over 1958–2000 slightly reduced the force of human infection with *T*. *cruzi* whereas the large-scale insecticidal campaigns implemented from 1966 onward drastically cut transmission without reaching its complete interruption [24]. Recently, a systematic review of household-centered interventions concluded that modifications of housing structure “had no impact on the control of vectors” of Chagas disease [65].

Our follow-up shows that this mainly Qom population comprised three subgroups of households that remained rather invariant over time: non-movers (~80%), with permanent residential stability; movers (~12%), households with substantial short-distance residential mobility within Area III, which usually demolished the old house; and other population losses (e.g., permanent out-migrants, ~8%). The main drivers for local movement were gaining better access to electricity, potable water, health care and education, better lands to cultivate [40], and cultural practices, such as dismantling a house after a family member passed away [66]. These mobility patterns are also consistent with ancestral practices: the Qom people originally included many semi-nomadic hunter-gatherer groups that built simple dwellings using available materials such as tree branches and animal skins [66, 67]. Several developments at the onset of the 20th century forced them to settle down [67], which severely affected their lifestyle and housing characteristics. We have also recorded a rural-to-peri-urban movement stream for the same reasons stated above combined with dwindling agriculture and local employment, leading to the creation of peri-urban slums within the municipality [68] and a strong dependency on welfare support.

In our study, household mobility was not random, as movers were more disadvantaged than non-movers [40]: most of the new houses built by movers and in-migrants were precarious, with unplastered mud walls and a metal-sheet roof. Baseline-infested houses (likely representing a higher-risk group) were demolished at a consistently slightly greater rate than non-infested ones, which may be relevant for long-term vector control. Similarly, post-spraying foci occurred more often in non-stable houses occupied by movers and out-migrants than in stable houses occupied by non-movers (Table 5), although the paucity of post-spraying foci strongly diminishes the power of any statistical test. Non-stable houses tended to spatially overlap with greater baseline infestation and subsequent high density of housing improvements (Fig 5). Most households (˃80%) owned the land they occupied under individual, familial or communal regimes [40], which gave them enormous flexibility to move within the area. In contrast, land tenure security increased the propagation of house infestation in other peri-urban settings, where the mobility patterns herein described were not observed [46]. The specific type of population movement described here was not recorded in a recent review of the subject [43]. Houses were frequently re-built in the proximities of their former location in a rural village of Bahia, Brazil [44], which suggests this practice may neither be strictly related to indigenous groups nor be uncommon. As expressed 25 years ago and still valid, “Shifts in the rates of new construction and demolished housing have never been explored in their relation to local household infestation in the literature” [69]. It is precisely this feature of mobility which may strengthen the effects of insecticide spraying, leading to the sustained impact observed.

Household relocation and associated housing dynamics may exert large negative impacts on the resident triatomine population through habitat destruction and host disappearance, but it may also create new suitable habitats free from insecticide; the latter can readily become infested through passive transportation of triatomines hidden in boxes and other belongings. This mechanism may explain why the prevalence of house infestation with *T*. *infestans* increased with the frequency of demolished houses and of new houses over two study periods across a large region of Minas Gerais, Brazil [69]. In contrast, following an effective insecticide spraying campaign (as in Area III), the net effects of household mobility within the study area and construction of new houses with local materials (albeit precarious) can be safely assumed to be detrimental to triatomine populations. A key point is that the inflow of migrants from other possibly infested areas outside of Pampa del Indio remained marginal over the seven-year period.

The high treatment coverage achieved in the community-wide insecticide campaign virtually suppressed house infestation and averted the occurrence of untreated houses as residual sources of triatomines except in one case. This is important because untreated urban dwellings have been identified as important sources in Arequipa, Peru [70]. Although the close supervision of spraying operations may have contributed to an enhanced performance of spray teams, the same intervention procedures achieved inferior results in other rural sections within Pampa del Indio municipality. In the context of household mobility and housing improvements described above, the sustained follow-up with selective sprays of the few detected foci led to the apparent suppression of house infestation at 78 MPS.

The three infested houses detected at 10 MPS were not residual foci in a strict sense since they had not been sprayed at the 2008 community-wide insecticide spraying (S2 Table, footnotes 3, 4). However, two of them had been sprayed with pyrethroids by healthcare agents two months before the insecticide campaign, and therefore qualified as residual foci of a slightly different nature. Several pieces of evidence do not support that the remaining post-spraying infestations originated from residual foci: pre-spraying and post-spraying infestations were not significantly associated, unlike in Areas I and II [37, 38]; wing shape analyses of *T*. *infestans* populations from Area III indicated the post-spraying foci were more likely related to external sources (not within Area III) than to baseline populations, with a low rate of exchange between Area III and Area I triatomines [41]; the protracted time gap between pre- and post-spraying foci at the same house, and the stage distribution and abundance of post-spraying foci (mainly including incipient bug colonies with a few adults and early-instar nymphs). Peripheral or sylvatic sources of *T*. *infestans* were very unlikely, as explained above. Barring the few putative cases of passive or active dispersal around a suspect source inside Area III, the ultimate origins of some post-spraying foci remain ill-defined.

Most post-spraying foci occurred in domestic premises with high scores for refuge availability and in households with very low educational level (average, 4.6 yr), as in the baseline survey. Half of the 10 post-spraying infested houses were Creoles’ (though they represented 10% of all households); most had brick-and-cement domiciles (60%), a low overcrowding index, no poultry inside domiciles (70%), few peridomestic structures, reportedly applied domestic insecticides (60%), and were not located in highly-infested communities at baseline (i.e., no neighborhood effect). The application of low-concentration insecticides in Area III is more likely related to householders’ socio-economic status and attitude responses rather than to having a direct impact on domestic triatomine populations [40]. Taken together, these features point to households with low-risk attributes at baseline [40], which paradoxically concentrated most post-spraying foci. While this inability to predict house reinfestation from preintervention models reflects imperfect system knowledge, they also suggest that other underlying (not readily observable) processes may have generated the new foci (e.g., passive transport from external sources).

Household-based vector surveillance corroborated the very low levels of post-spraying infestation shown by timed-manual searches, and revealed the invasion of four other triatomine species. Householders’ notifications of triatomines performed better than timed searches during the initial vector surveys but not later, suggesting the residents’ motivation may wane over time and when non-target triatomine species prevail. While these results reveal the potential of community-based efforts for sustainable vector surveillance, strengthening local capacities and periodic stimulation of community involvement are crucial [3, 71]. The widespread network of local healthcare workers (most of Qom descent) who assisted in this task is a major local asset for vector and disease surveillance [72], and provides a means to resolve the cultural barriers that often obstruct health interventions in indigenous communities [73].

Despite the virtual suppression of *T*. *infestans* after insecticide spraying, none of the sylvatic triatomine species (not targeted for control) were able to recolonize peridomestic habitats except *T*. *sordida*, as in Area I [74], and all failed to establish domestic colonies. The triatomines collected by householders were spatially aggregated and mainly occurred in one community. Whether the surrounding landscape facilitated the invasion of sylvatic triatomines [75] or local residents were more aware and committed to vector surveillance, perhaps boosted by the contagious nature of community participation [76], remains for future investigation.

The baseline populations of *T*. *infestans* displayed evidence of moderate pyrethroid resistance throughout Area III, thus predicating the subsequent occurrence of vector control failures as in Areas I and II [37, 38]. One highly persistent bug population re-treated four times in Area I displayed a mean resistance ratio (RR) of 7.2 [37], whereas another persistent population in Area II displayed reduced mortality (60–70%) in the screening tests [38]. In contrast, pyrethroid applications in Area III were highly effective and no sprayed focus persisted over several surveys. Why did post-spraying infestation rates differ so much from those expected on the basis of diagnostic screening tests and background evidence is unclear. One potential explanation is that diagnostic mortalities ranging from ~30 to 70% and RR from ~8 to 20 are inconsistent predictors of pyrethroid effectiveness in real-life rural settings (i.e., fall in a diagnostic indeterminate zone), whereas RR>50 are invariably associated with vector control failures in northern Argentina and Bolivia [51, 77–79]. Another, not mutually exclusive, explanation is that low-density triatomine populations subject to removal by timed searches followed by insecticide spraying were likely decimated and then faced the additional constraints imposed by environmental and demographic stochasticity. The substantial number of triatomine populations screened for pyrethroid resistance and evidence of its widespread occurrence at moderate levels minimized the issues related to sample size and representativeness [80]. Our results also reveal the importance of simultaneous monitoring of house infestations, insecticide resistance and other social determinants (housing, mobility) to assess whether post-spraying infestation indices match the outcome of screening tests or other underlying causes for vector control failure [77] or success affect the outcomes, as in the current study.

The geographic distribution of pyrethroid resistance in *T*. *infestans* is concentrated in central areas of the Gran Chaco region [81]. In Argentina, the main hotspot of pyrethroid resistance is close to Pampa del Indio [79]. The limited flight dispersal of *T*. *infestans* and the reduced exchange of household goods between resource-constrained rural areas (linked to passive transport of triatomines) may have hampered the expansion of pyrethroid-resistant foci. Substantial population structuring in *T*. *infestans* [41, 82, 83] may also contribute to explain household-level variations in resistance status [84]. Therefore, the combination of several factors may help explain the heterogeneous impact of the same intervention protocol among Pampa del Indio study areas.

Our study had some limitations not described above: i) the limited sensitivity of timed-manual searches to detect triatomine infestation, especially at low densities [85], was in part compensated by repeated searches over the follow-up and household-based vector surveillance. Similarly, the low (38 MPS) or nil (59 MPS) house infestation levels revealed by these partial vector surveys were confirmed subsequently by full-coverage vector survey; ii) the refuge availability index showed low variability in domiciles, which may difficult the detection of changes associated with housing improvements; iii) the limited number of female triatomines at a given focus precluded the assessment of pyrethroid RR and testing the association between resistance status and infestation at household level; iv) the very few post-spraying foci detected impeded us from conducting a detailed risk factor analysis and examine whether the house infestation risk models developed from baseline data had any predictive power, and v) the destination of many out-migrant households frequently included missing data due to the difficulties in obtaining information for those who left the area.

### Implications for vector control and research

Both household mobility and housing quality are key to understand house infestation dynamics in the context of vector control interventions and demographic change, and the details of their interaction with other factors matter. For example, whether a vacant house is immediately re-occupied by newcomers (rather than being torn down), or whether movers always build the new house from scratch will determine the fate of pre-existing infestations; so does whether housing improvements are combined with insecticide spraying or not. These aspects pose additional challenges to traditional housing and vector control programs since for instance, many sprayed (presumably protected) houses disappear while new (unprotected) houses built with used or new materials emerge. The relationship between housing instability, household mobility and infestation requires further investigation in different endemic and cultural settings. Underlying these patterns, our study discloses a chronic housing crisis in rural areas of the Argentine Chaco, tightly linked to one of the goals in the 2030 Agenda for Sustainable Development: ensure access for all to adequate, safe and affordable housing and basic services.

Which vector surveillance strategy is appropriate for scenarios such as those in the rural Chaco? In the context of low house infestation levels after interventions over extended, at times inaccessible areas, annual house searches for triatomines are neither cost-effective [10, 86, 87] nor sustainable [8]. One of the challenges vector control programs face there is how to sustain the initial progress in the face of their limited operational capabilities and the competing demands posed by recurring outbreaks of dengue and other mosquito-borne diseases [4]. Mixed approaches including community-based vector surveillance showed promising results [5, 33, 34, 36, 72, 86–88]. Early detection of house infestation with triatomines and prompt insecticide treatment can be achieved through coordinated local efforts among householders, the primary healthcare system and other grassroots organizations, especially when rural communities are disperse and access is difficult. School-based health education interventions may assist in fostering community participation in vector surveillance and control. A crucial point in such community-based programs is related to providing an appropriate response to householders’ notifications (service delivery): who will provide gear, insecticide and apply it properly [5, 88]. The implementation, maintenance and supervision of the vector surveillance-and-response system are essential for long-term disease control in high-risk contexts [20, 88, 89]**.**

## Supporting information

S1 Text Detailed results of building characteristics of baseline-infested and newly-built houses, and householders’ triatomine collection during the surveillance phase in Area III of Pampa del Indio, 2008–2015. (DOCX)

S1 Checklist STROBE Checklist. (DOC)

S1 Fig Spatial distribution of post-spraying house infestation with *T*. *infestans* and householders’ collections of other triatomine species in Area III of Pampa del Indio, 2008–2015. (TIF)

S2 Fig Improved house with brick-and-cement walls, cement floor, corrugated metal-sheet roof and wooden ceiling showing numerous fecal smears of triatomine infestation in Area III of Pampa del Indio. (TIF)

S1 Table Distribution of house unit status in Area III of Pampa del Indio, 2008–2015. (DOCX)

S2 Table Association between baseline (0 MPS) and post-spraying (10–78 MPS) house infestation with *Triatoma infestans*, as determined by timed-manual searches in Area III of Pampa del Indio, 2008–2015. (DOCX)

S3 Table Longitudinal data including house infestation, environmental and sociodemographic variables in Area III of Pampa del Indio, 2008–2015. (XLSX)
