# Supplementary material for: Beating the odds: Sustained Chagas disease vector control in remote indigenous communities of the Argentine Chaco over a seven-year period
Source: PLoS Negl Trop Dis. 2018 Oct 2;12(10):e0006804. doi: 10.1371/journal.pntd.0006804 (PMC6168123; doi:10.1371/journal.pntd.0006804)
Supplement: S1 Text — (DOCX) [file pntd.0006804.s001.docx]

*Building characteristics of baseline-infested and newly-built houses*

Among the baseline-infested houses that were permanently occupied (stable), the proportion with brick-and-cement walls steadily increased from 24.7% at baseline to 44.6% at 78 MPS (χ*^2^* = 15.2, df = 2, p = 0.001). Among stable houses with mud walls at baseline, only 11.1% and 8.5% were improved by 49 and 78 MPS, respectively.

Of the 236 new housing units registered during the follow up, 64.0% had exclusively mud-walled domiciles whereas 16.1% had brick-and-cement walls, although a fraction of them also had an additional mud-walled domicile. Among the 151 new houses with mud-walled domiciles, the majority had unplastered walls (58.3%), dirt floors (92.7%), and tarred-cardboard roofs (32.5%). Among the 38 brick-and-cement new houses, most had plastered walls (52.6%), corrugated metal-sheet roofs (86.8%) and cement floor (71.1%). Of all brick-and-cement new houses, 39.5% were provided by the government-sponsored housing program, and always included plastered walls, corrugated metal-sheet roofs, and cement floors. Only 1 (5%) of the 20 new houses with brick-and-cement walls were built by the housing program over 2008-2012 whereas 14 (77.8%) of 18 were built over 2012-2015.

Table. Building characteristics (walls, roof, floor) of newly-built houses in Area III of Pampa del Indio, 2009-2015.

| Section | Material | % (no. of houses) | | | |  |
| --- | --- | --- | --- | --- | --- | --- |
|  |  | Walls | | | | Total |
|  |  | Mud | Brick-and-cement | Other | NR* |  |
| Roof | Cardboard | 32.5 (49) | 7.9 (3)^1^ | 30.8 (4) | 0.0 (0) | 56 |
|  | Tin metal | 63.6 (96) | 86.8 (33) | 46.2 (6) | 0.0 (0) | 135 |
|  | NR* | 4.0 (6) | 5.3 (2) | 23.1 (3) | 100.0 (34) | 45 |
| Plastered walls | Yes | 33.8 (51) | 52.6 (20) | 7.7 (1) | 0.0 (0) | 72 |
|  | No | 58.3 (88) | 39.5 (15) | 76.9 (10) | 0.0 (0) | 113 |
|  | NR* | 7.9 (12) | 7.9 (3) | 15.4 (2) | 100.0 (34) | 51 |
| Floor | Dirt | 92.7 (140) | 15.8 (6)^1^ | 76.9 (10) | 0.0 (0) | 156 |
|  | Cement | 3.3 (5) | 71.1 (27) | 0.0 (0) | 0.0 (0) | 32 |
|  | NR* | 4.0 (6) | 13.2 (5) | 23.1 (3) | 100.0 (34) | 48 |
| Total |  | 64.0 (151) | 16.1 (38) | 5.5 (13) | 14.4 (34) | 236 |

^1^ Both the cardboard roof and dirt floor were associated with an additional mud-walled domicile.

NR*: Not registered data

*Householders’ triatomine collection during the surveillance phase*

Householders collected 45 triatomines (including 23 males, 17 females and 5 nymphs) at 25 different houses and other Reduviidae bugs (not Triatominae) at 15 houses over the follow-up. The former included 8 *T. infestans* (at 3 houses, previously positive by timed searches), 21 *T. sordida* (at 12 houses), 12 *Panstrongylus geniculatus* (at 10 houses), 3 *Triatoma platensis* (at 3 houses), and 1 *Psammolestes coreodes* (at 1 house); the last three species were never collected by timed searches. Bug collections most frequently included 1-2 adult triatomines or Reduviidae per house around domestic premises (i.e., veranda, window screens, domestic lights). Householders’ collections were significantly aggregated at a global scale beyond 800 m, and were mainly located in one community (S1 Fig).
