# Supplementary figures and images for: Beating the odds: Sustained Chagas disease vector control in remote indigenous communities of the Argentine Chaco over a seven-year period
Source: PLoS Negl Trop Dis. 2018 Oct 2;12(10):e0006804. doi: 10.1371/journal.pntd.0006804 (PMC6168123; doi:10.1371/journal.pntd.0006804)

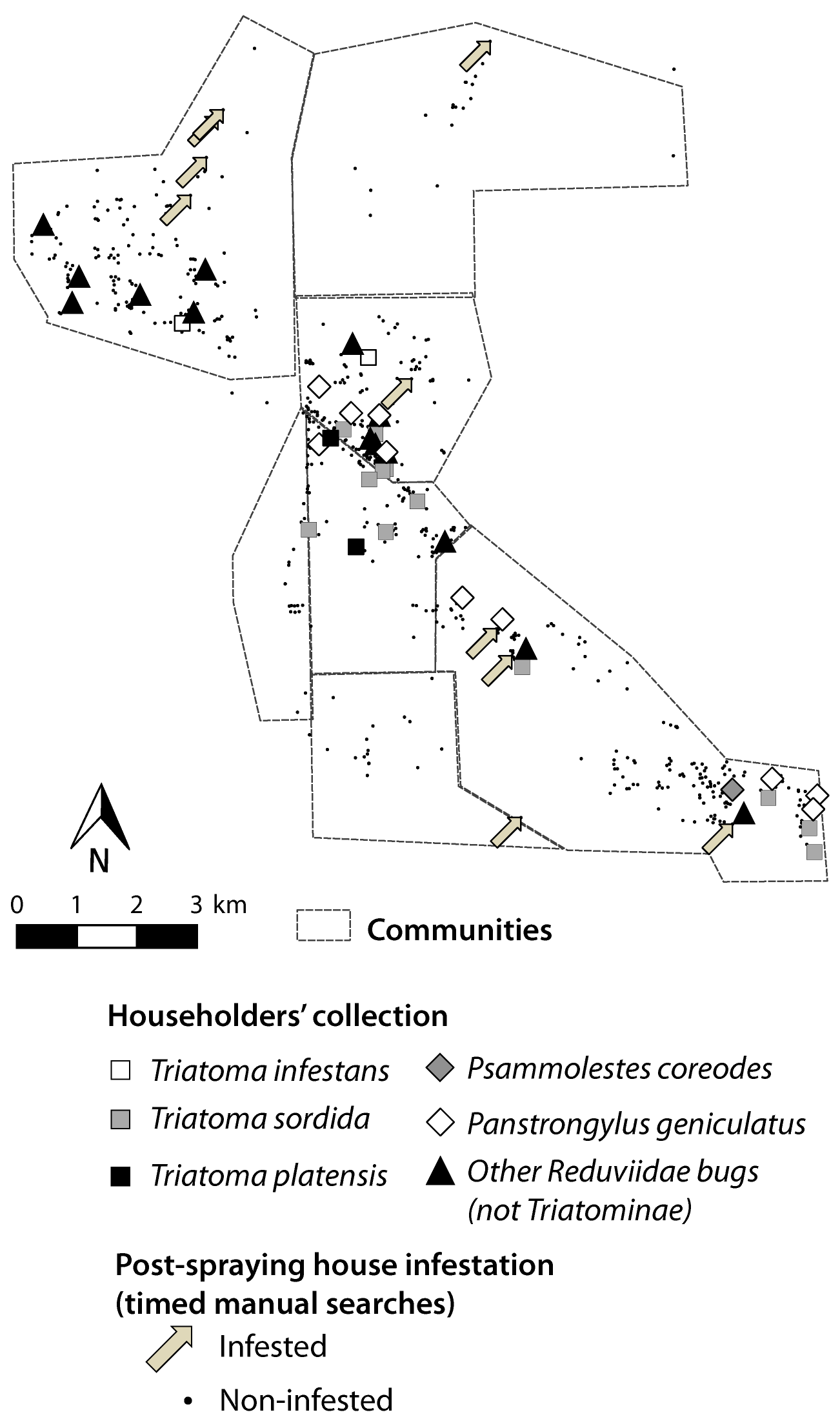

Supplement: S1 Fig — (TIF) [file pntd.0006804.s003.tif]

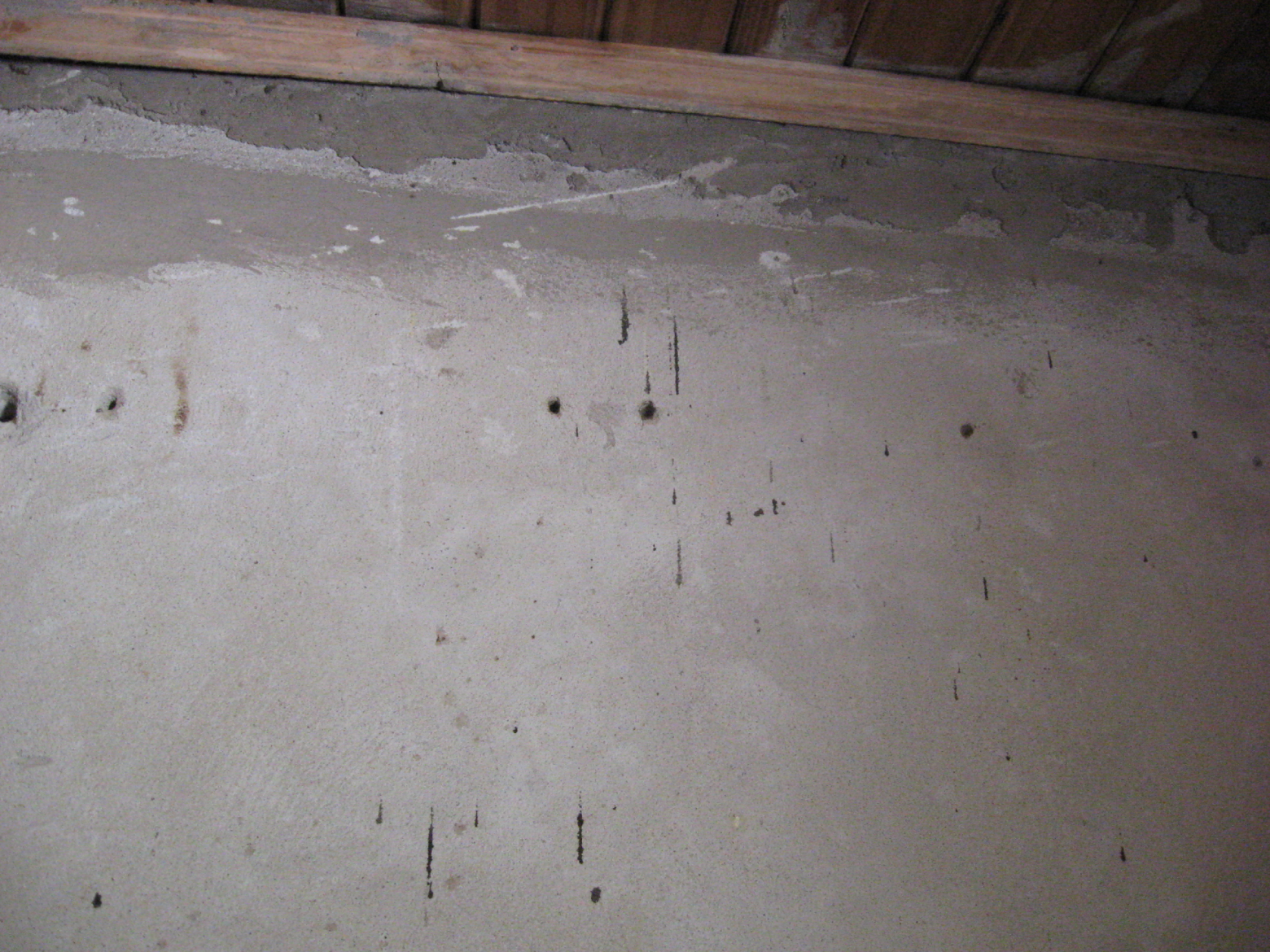

Supplement: S2 Fig — (TIF) [file pntd.0006804.s004.tif]
