# Supplementary material for: Beating the odds: Sustained Chagas disease vector control in remote indigenous communities of the Argentine Chaco over a seven-year period
Source: PLoS Negl Trop Dis. 2018 Oct 2;12(10):e0006804. doi: 10.1371/journal.pntd.0006804 (PMC6168123; doi:10.1371/journal.pntd.0006804)
Supplement: S1 Table — (DOCX) [file pntd.0006804.s005.docx]

| House status | | No. of house units (No. inspected or %)* | | | | |  |
| --- | --- | --- | --- | --- | --- | --- | --- |
|  |  | 0 MPS | 10 MPS | 18 MPS | 49 MPS | 78 MPS |  |
| Occupied | Inspected^1^ | 389 (96.3) | 401 (95.0) | 391 (94.4) | 416 (95.0) | 420 (93.8) |  |
|  | Closed^2^ | 15 (3.7)^4^ | 20 (4.7) | 19 (4.6) | 15 (3.4) | 21 (4.7) |  |
|  | Rejected^3^ | 2 (0.5) | 1 (0.2) | 5 (1.2) | 7 (1.6) | 7 (1.6) |  |
|  | Subtotal | 404 | 422 | 414 | 438 | 448 |  |
| Vacant |  | 7 (4)^5^ | 14 (6) | 32 (14) | 33 (13) | 37 (13) |  |
| Total |  | 411 | 436 | 446 | 471 | 485 |  |
| New | | - | 29 (6.9) | 26 (6.3) | 86^6^ (19.6) | 89 (19.9) |  |
| Demolished |  | - | 5 (1.2) | 17 (4.0) | 43 (10.4) | 64 (14.6) |  |

**S1 Table. Distribution of house unit status in Area III of Pampa del Indio, 2008-2015.**

* In total, 86 houses were inspected at 38 MPS (2011), including 25 where householders reported bug presence; 23 houses were inspected at 59 MPS (2013), including four where householders reported bug presence only and eight who suspected a house infestation.

** Seventeen public buildings were registered, and 6, 1, 4, 1 and 3 of them were inspected for infestation over the follow-up, respectively.

^1^ Occupied house units inspected for triatomines by timed-manual searches.

^2^ House residents were absent during inspection rounds.

^3^ House residents refused to allow the search of premises for triatomines.

^4^ Includes one occupied house not inspected due to its remote location.

^5^ Only includes vacant houses at baseline that were subsequently occupied during the follow-up. Ten vacant houses at baseline demolished during the follow-up were excluded.

^6^ Four new house units registered at 38 MPS and demolished at 49 MPS were taken to be occupied at 49 MPS and subsequently demolished by 78 MPS in order to be included among the surveyed houses.

All houses: 422.77 + 0.8718*MPS, df 4, adj R^2^ = 0.8904, P = 0.01

Occupied: 408.79 + 0.5292*MPS, df 4, adj R^2^ = 0.8746, P = 0.01

New houses: 17.43 + 1.0340*MPS, df 3, adj R^2^ = 0.7899, P = 0.07

Vacant: 13.98 + 0.3426*MPS, df 4, adj R^2^ = 0.5826, P = 0.08

Demolished: -0.54 + 0.8461*MPS, df 3, adj R^2^ = 0.9903, P < 0.01
