# Supplementary material for: Beating the odds: Sustained Chagas disease vector control in remote indigenous communities of the Argentine Chaco over a seven-year period
Source: PLoS Negl Trop Dis. 2018 Oct 2;12(10):e0006804. doi: 10.1371/journal.pntd.0006804 (PMC6168123; doi:10.1371/journal.pntd.0006804)
Supplement: S2 Table — (DOCX) [file pntd.0006804.s006.docx]

| Baseline house infestation | Post-spraying house infestation | | | | | | |  |
| --- | --- | --- | --- | --- | --- | --- | --- | --- |
|  | Yes (%)^1^ | No | | No data | | Total | |  |
| Yes | 3 (2.4) | | 116 | | 4^2^ | | 123 | |
| No | 4 (1.5)^3^ | | 256 | | 7^2^ | | 267 | |
| No data | 3 (1.2)^4^ | | 241 | | 10 | | 254^5^ | |
| Total | 10 (1.6) | | 613 | | 21 | | 644 | |

**S2 Table. Association between baseline (0 MPS) and post-spraying (10-78 MPS) house infestation with *Triatoma infestans*, as determined by timed-manual searches in Area III of Pampa del Indio, 2008-2015.**

^1^ Percentage relative to the number of inspected houses at each category of baseline infestation, excluding houses with no data.

^2^ Demolished after the baseline survey.

^3^ Two houses positive at 10 MPS, which had been sprayed with insecticides by local healthcare workers two months before the baseline survey, when they were negative by timed searches, were not re-sprayed at 0 MPS. Two other houses were positive for the first time at 49 MPS.

^4^ Neither inspected for triatomines nor sprayed with insecticides at baseline: one was a new house built after community-wide spraying; another one was remote and could not be accessed during the insecticidal campaign, and the remainder was closed at baseline, negative at 10 MPS, and infested at 18 MPS.

^5^ Most (91.2%) houses were built after the baseline survey.
